# Chinese patients’ response to doctor–patient relationship stimuli: evidence from an event-related potential study

**DOI:** 10.1186/s40359-022-00961-y

**Published:** 2022-11-05

**Authors:** Zehan Ye, Jiaoyan Pang, Wei Ding, Wen He

**Affiliations:** 1 Department of Psychology, Shanghai Normal University, Shanghai, 200234 China; 2 School of Government, Shanghai University of Political Science and Law, Shanghai, China

**Keywords:** Doctor–patient relationship, Event-related potential, P2, LPP

## Abstract

**Background:**

With improvements in medical technology, the doctor–patient relationship should be further improved. However, disputes between doctors and patients have increased, with the two groups frequently hurting each other. Therefore, we sought to explore the perception of Chinese patients regarding the stimuli of doctor–patient relationships with different valence.

**Methods:**

We used event-related potential (ERP) to explore the brain electrical activity of 19 undergraduate participants who had a clinical experience in the previous 6 months where they perceived negative, neutral, and positive doctor–patient relationships. The ERPs were recorded, and the early ERP components (P2) and late positive potential (LPP) were measured.

**Results:**

Compared with the stimuli of negative doctor–patient relationships, those of positive doctor–patient relationships would attract more attention and have larger P2 amplitude; LPP was larger for the stimuli of negative doctor–patient relationships than neutral ones in the 500–800 ms, while in the 1100–1500 ms, the stimuli of neutral doctor–patient relationships elicited larger LPP amplitude than positive ones.

**Conclusion:**

Patients paid more attention to the stimuli of positive doctor–patient relationships because they expected to have the same positive relationship. Although threatening elements in negative doctor–patient relationships would catch patients’ attention and make them have implicit emotional regulation, neutral stimuli with poker-faced doctors would cause lasting attention. These results illustrate the patients’ real perception of the different valence of doctor–patient relationship stimuli.

**Supplementary Information:**

The online version contains supplementary material available at 10.1186/s40359-022-00961-y.

## Introduction

The doctor–patient relationship is established from the exchange of medical treatment between doctors and patients [6]. This relationship should be harmonious and trustworthy in China, and with the improvement of medical technology, it should be further improved. However, recent research has indicated that disputes between doctors and patients have increased, with the two groups hurting each other frequently [55]. A *White Book on the Practice of Chinese Doctors* showed that 62% of doctors and patients had medical disputes of varying degrees (Chinese Medical Doctor Association) [9], and some scholars have found that patients widely consider the current doctor–patient relationship to be poor [31, 38].

When patients confront positive doctor–patient relationships, they have confidence in doctors, and the positive relationships make their emotions stable [5, 24, 52]; however, if they are met with negative ones, they not only show discontent with the hospital and doctors but also reduce compliance with the doctor’s advice [13]. Some patients even believe that it is appropriate to express anger through violent behavior when faced with negative doctor–patient relationships [12]. In other words, when patients face doctor–patient relationships with different valence, they show contrasting reactions. Nevertheless, previous research has been conducted in the form of interviews and questionnaires, and patients’ perceptions of different valences of doctor–patient relationship stimuli when confronted with them are not yet known.

From the view of stimuli perception, individuals had psychological differences in processing stimuli of different valence: participants would blink when watching the negative stimuli subconsciously in the fast–presenting task instead of the neutral ones [1, 39]. This means that negative stimuli would be processed more efficiently and catch human attention. Likewise, individuals become more aware of the details of positive stimuli when compared with neutral ones [3, 15]. These studies suggest that individuals would have an attentional bias to negative or positive stimuli compared to neutral stimuli. This phenomenon is also in line with the theory of motivated attention, which posits that human attention is regarded as an information-processing process involving the selection and evaluation of input related to motivation [29]. The theory suggests that humans perceive various types of stimuli every day—some with motivational information, some with emotional valence, some with social signaling, and so on [29, 44]. Among them, stimuli with emotional valence attract individual attention, while those with motivational valence sustain attention for a longer time [4, 46]. It seems that patients perceive the doctor–patient relationship differently because the related stimuli have different motivational messages for them,however, previous studies have not indicated how patients are motivated to perceive such a relationship. Therefore, we chose event-related potential, which could help us understand the real perceptions of patients more accurately, to investigate the different physiological responses in patients (Additional file 1: Fig. S1).

Regarding event-related potential components, P2 has been associated with early perceptual processes and responded to stimuli of different valences in diverse ways [11]. Kanske and Kotz [26] found that positive words had greater P2 amplitude than neutral words in the lexical decision task. In addition, scholars have found that when participants watch negative words and pictures, they have a larger P2 amplitude [27]. Both negative and positive stimuli elicit P2 amplitude, but negative stimuli elicit greater amplitude than positive and neutral ones [10, 22]. In addition, LPP could reflect the processing of motivational stimuli [37], and it can also be elicited at different amplitudes by stimuli of disparate valence. Prior studies have shown that compared with neutral stimuli (e.g., scenery), negative and positive stimuli could elicit larger LPP amplitude and that negative stimuli would elicit larger LPP amplitude than positive and neutral ones [16, 19, 42]. Some scholars [14] have found that both task- and emotion-related images could cause an increase in LPP amplitude, but the amplitude of emotion-related induction was larger. They suggested that this was related to the motivation of the perceivers [14, 29], that is, the stimulus related to individual motivation would trigger the individual’s attention (e.g., food stimuli). Arguably, P2 and LPP could be used to provide a more comprehensive account of individuals’ perceptions when the stimuli are provided from emergence to disappearance.

Based on extant studies on Chinese doctor–patient relationships, stimuli of doctor–patient relationships with disparate valence were used to explore the patients’ actual perceptions. It could be said that patients have different behavioral reactions when they perceive doctor–patient relationships with different valence. When perceiving stimuli with different valence, they have different perceptions due to motivated attention, which is reflected in their physiological indicators. To understand patients’ real response to doctor–patient relationships, the specific objective of this study was to investigate their EGG response when perceiving stimuli of doctor–patient relationships of different valence. Based on the results of studies on stimuli perception and the theory of motivated attention, we propose the following two hypotheses: (1) compared with the neutral doctor–patient relationship stimuli, positive and negative ones attracted more attention and generated a larger P2 amplitude; (2) doctor–patient relationship stimuli with different valence elicited different LPP amplitudes. Compared with the stimuli of positive doctor–patient relationships, the perception of the stimuli of negative doctor–patient relationships will elicit larger LPP amplitudes and make patients pay more attention.

## Material and methods

### Participants

Nineteen right-handed participants from Shanghai Normal University (10 males, nine females; age *M* = 19.26, *SD* = 1.15) with no history of neurological disorders, brain injuries, or developmental disabilities participated in the current experiment. All participants had a normal or corrected-to-normal vision and had had a clinical experience in the previous 6 months. This study was approved by the local ethics committee of Shanghai Normal University, and informed consent was obtained from all the participants at the beginning of the experiment.

### Materials and design

We divided the stimuli of doctor–patient relationships into three categories (positive, neutral, and negative), all of which were extracted from 190 colored pictures of doctor–patient relationships of different valences taken by professional psychological groups. The use of an image processing software showed that all stimuli were similar in pixel count and size. Specifically, the images had a size of 433 × 320 pixels and a resolution of 300 dpi. A total of 34 psychology graduate students were asked to evaluate the pictures to identify their valence in expressing doctor–patient relationships of different valence. All pictures were rated on a 7-point scale ranging from 1 (“very negative”) to 7 (“very positive”). We averaged the scores of each picture after they were evaluated by graduate students and compared the scores with the average number 4 using a single sample t-test. Pictures whose scores were not significantly different were classified as neutral. Pictures with high scores (> 6) were classified as positive stimuli, while those with low scores (less than 2) were classified as negative stimuli. Furthermore, pictures with blurred movements or similar characters or expressions were removed to improve discrimination. Thirty stimuli were included in each category. The deviation between the average scores of the positive and negative stimuli and the average score of the neutral stimuli was no greater than 2. Repeated measures analysis of variance (ANOVA) was performed for the mean score of each type of picture [positive stimuli (6.05 ± 0.31), neutral stimuli (4.15 ± 0.23), and negative stimuli (1.90 ± 0.59)], and significant differences were observed in the three categories, *F* (2, 58) = 814.78, *p* < 0.001. Therefore, the pictures used in the experiment reflect doctor–patient relationships of different valence.

### Experimental procedure

Participants sat alone in a quiet room during the recording. A 14-inch color monitor was placed in front of them, and the stimuli were presented in the center of a white background via E-Prime 3.0. First, the participants were required to use their imagination for 2 min at the beginning of the experiment under the guidance of the experimenter; that is, they were asked to imagine the process of going to a hospital as a patient, and their behavior, clothes, and communication with a doctor, to the best of their abilities. In the next part, they were requested to record their images on a computer to deepen their perception of the patients’ identities. After recording their role as patients, they were asked to perceive the stimuli by giving each one a score.

**Fig. 1:**
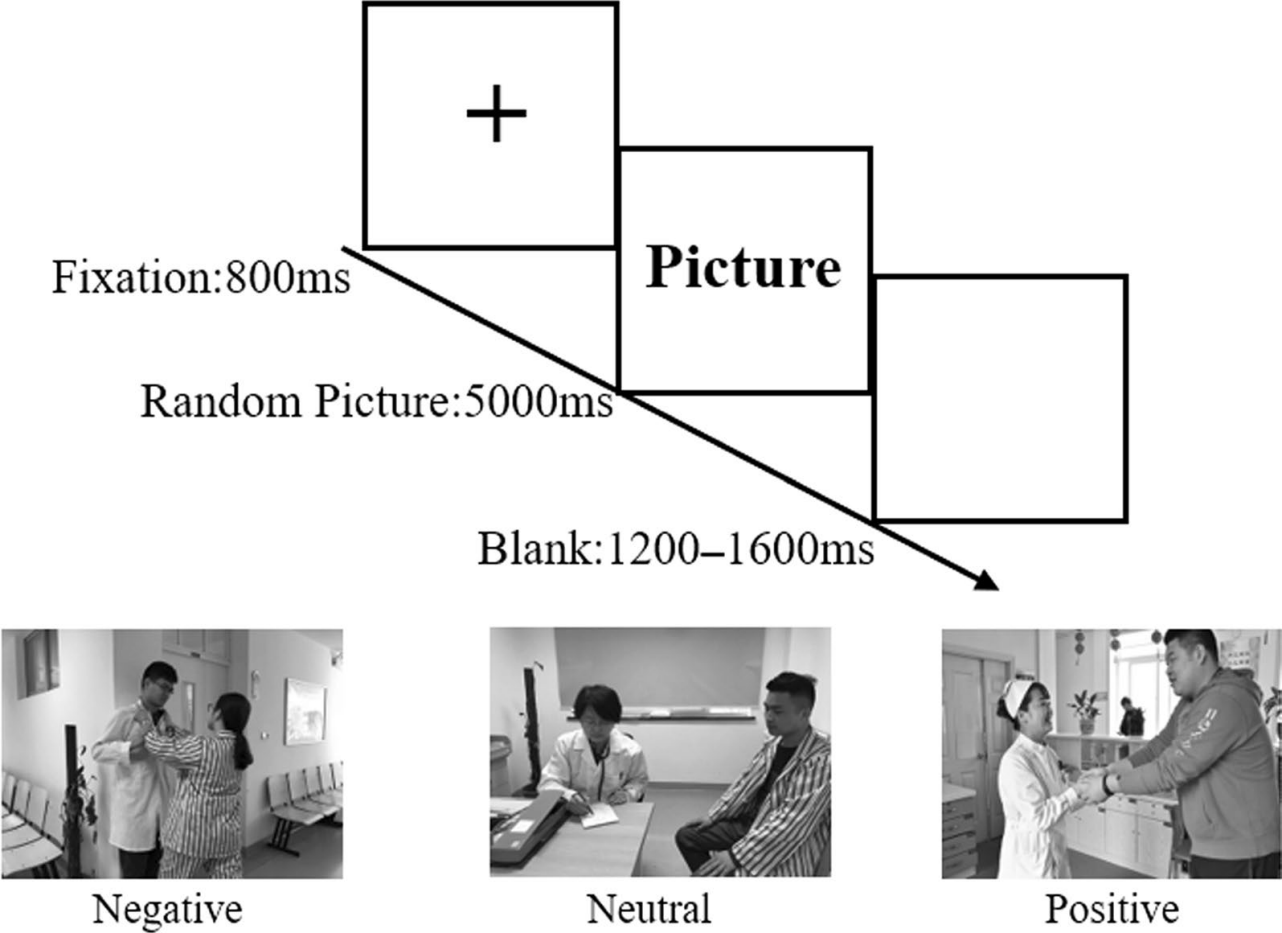
Procedure of the experiment

The participants were instructed to perceive the stimuli before the formal experiment by considering the quality of doctor–patient relationships and to score them on a scale from 1 to 3 stimuli (1 = negative, 2 = neutral, and 3 = positive). They were then asked to complete a set of practice routines that included four stimuli in each category, which would no longer be used in the formal experiment. Patients could choose to start the formal experiment when they felt that they were familiar with the experimental rules, and the device recorded the EEG. A fixation was presented on the white screen for 800 ms in each trial of the formal experiment, followed by a positive, neutral, or negative stimulus displayed in random order in the center of the screen for 5000 ms. The interval between the end of the different stimuli and the onset of the next fixation point was a random time from 120 to 1600 ms (Fig. 1). The program automatically accessed the next trial after the time limit was exceeded, regardless of whether the participants had perceived the stimuli. Four blocks were used, with each block containing 45 trials and a minimum 30-s rest between blocks. In total, 180 trials were conducted. Each stimulus was randomly repeated twice, and the entire recording lasted approximately 25 min. The Brain Products analysis system was used to sort and analyze the data after the experiment.

### Recording and analysis

The EEG was recorded from a 64-electrode scalp cap using a 10–20 system (Brain Products, Munich, Germany). Channels Tp9 and Tp10 were used as references during the recording, and the contact point was the midpoint between the Fp1 and Fp2 electrode points. The electrooculogram (EOG) was recorded using electrodes placed on the outer canthi of the right eye and below the left eye. Offline, EEG signals were referenced to the average of the left and right mastoids. EOG artifacts were corrected using an independent component analysis [25]. All the electrode impedances were maintained below 10 kΩ. The EEG and EOG activities were amplified at 0.01–100 Hz bandpasses and sampled at 500 Hz. The recording was split into 1700-ms epochs, beginning 200 ms before stimulus onset. Epochs with amplitude values exceeding ± 80 μV at any electrode were excluded.

This experiment focused on the attention phase of the selection of emotional information; thus, the early component—that is, P2—was analyzed [28]. Subsequently, we analyzed LPP, which reflects stimulus perception and emotional experience [19]. P2 is a positive component that is quantified in the central frontal region with a peak latency of approximately 200 ms, and LPP components exhibit the largest amplitude near the posterior midline of the scalp. Different sets of electrodes for each component were chosen based on the topographical distribution of grand-averaged ERP activity and previous studies. Analyses were conducted over the peak amplitudes of the P2 components and mean amplitudes of the LPP components in different time windows. Fz, Cz, and Pz were selected for the analysis of P2 (130–200 ms; [2, 50]), CP3, CPz, CP4, P3, Pz, and P4 were selected for the LPP analysis [8, 20, 33], which was divided into 300–500 ms, 500–800 ms, 800–1100 ms, and 1100–1500 ms time windows for measurement [47, 56]. For the peak amplitudes of the P2 components, a repeated measures ANOVA of 3 (type: positive/neutral/negative) × 3 (electrodes: Fz/Cz/Pz) was performed, while the LPP was subjected to a repeated measures ANOVA of 3 (type: positive/neutral/negative) × 4 (time windows: 300–500 ms/500–800 ms/800–1100 ms/1100–1500 ms) × 6 (electrodes: CP3/CPz/CP4/P3/Pz/P4).

## Results

### Behavioral data

We conducted a repeated measures ANOVA to test the differences in perception of doctor–patient relationship stimuli and found that the valence of these stimuli had a significant main effect, *F* (2, 36) = 5139.02, *p* < 0.001, ${\upeta }_{\mathrm{p}}^{2}$ = 0.997). Compared to the negative (*M* = 1.10, *SD* = 0.043) and neutral (*M* = 2.02, *SD* = 0.06) doctor–patient relationships, the score of the stimuli of positive doctor–patient relationship (*M* = 2.89, *SD* = 0.04) was the highest, and these stimuli were all significant (*p* < 0.001).

### Event-related potentials

#### P2

**Fig. 2:**
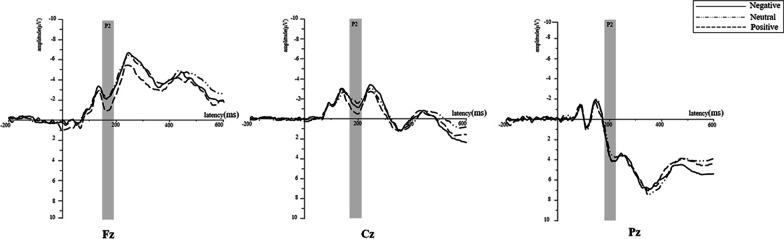
Ground average ERP: Fz, Cz, and Pz electrode sites were selected and analyze d P2 (130–200 ms). The gray color for P2 indicated an approximate range of the windows across all conditions

The main effects of stimuli, *F* (2, 36) = 4.23, *p* = 0.022, ${\upeta }_{\mathrm{p}}^{2}$ = 0.19, and electrodes, *F* (2, 36) = 16.06, *p* < 0.001, ${\upeta }_{\mathrm{p}}^{2}$ = 0.47, were significant. The interaction between the electrode and stimuli was not significant, *F* (4, 72) = 1.31, *p* = 0.273, ${\upeta }_{\mathrm{p}}^{2}$ = 0.07. From the posthoc comparisons, a marginally significant difference was observed between the positive and neutral doctor–patient relationship stimuli (*p* = 0.056; Fig. 2): the positive doctor–patient relationship stimuli (1.69 ± 3.45 μV) elicited a more positive P2 than the neutral ones (0.89 ± 3.62 μV).

#### LPP

**Fig. 3:**
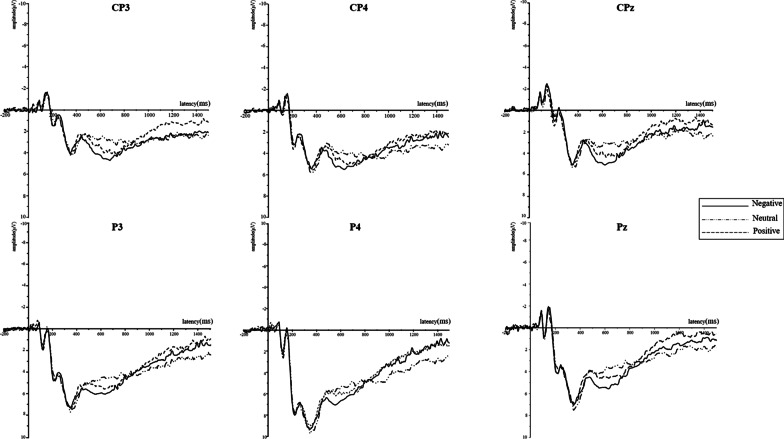
ERP waveforms of the late positive potential (LPP)

We observed LPP in four time windows. The main effect of the stimuli was insignificant, *F* (2,36) = 0.89, *p* = 0.421, ${\upeta }_{\mathrm{p}}^{2}$ = 0.05, and the main effects of time window, *F* (3,54) = 13.31, *p* = 0.001,${\upeta }_{\mathrm{p}}^{2}$ = 0.43, and electrode, *F* (5, 90) = 7.55, *p* < 0.001, ${\upeta }_{\mathrm{p}}^{2}$ = 0.30 were significant. The interaction of stimuli × time window was also significant, *F* (6,108) = 6.20, *p* = 0.001,${\upeta }_{\mathrm{p}}^{2}$ = 0.26. Post-hoc comparisons showed that negative doctor–patient relationship stimuli (5.08 ± 4.05 μV) elicited a larger LPP than neutral ones (3.88 ± 3.65 μV; *p* = 0.017) but were insignificant compared with positive ones (4.59 ± 3.53 μV) in the time window of 500–800 ms (Fig. 3). In the 1100–1500 time window, neutral doctor–patient relationship stimuli (2.65 ± 2.66 μV) elicited a larger LPP than positive ones (1.42 ± 2.19 μV; *p* = 0.044; Fig. 3) but were insignificant compared with negative ones (1.94 ± 2.96 μV).

## Discussion

The present study intended to explore patients’ responses when they perceived stimuli of doctor–patient relationships of different valence. In previous studies, both negative and positive stimuli elicited a larger P2 amplitude than neutral ones [36]. However, a marginally significant difference was observed between the stimuli of positive and neutral doctor–patient relationships in the present study. That is, compared to the stimuli of neutral doctor–patient relationships, those of positive relationships elicited a larger P2 amplitude, which was associated with attention distribution [28]. From media coverage, doctor–patient conflicts are more likely to be reported than positive events, suggesting that patients often come into contact with information about negative doctor–patient relationships [57]. However, for the stimuli of positive doctor–patient relationships, their elements could make the patient feel psychologically satisfied (e.g., the doctor’s concern for the patient; [40],therefore, the reason for this discrepancy might be that individuals paid increased attention to a positive doctor–patient relationship (Fig. 2). The change in P2 reflected that patients were expected to have a positive doctor–patient relationship and then showed attention to positive rather than negative ones in the early stage [30].

Perception reactions were generated in the participants’ brains after paying attention to the stimuli. The LPP reflects the conscious perception of emotional stimulation as it is considered an effective physiological indicator of emotion regulation [18, 19]. Previous studies on cognitive evaluation have shown that enhancing emotional experience leads to an increase in LPP [17, 18]; correspondingly, weakening emotional experience leads to a decrease in LPP [18, 43]. We found no difference in LPP in the 300–500-ms time window (Fig. 3), which means that patients may have consciously perceived and classified the stimuli [18, 35]. However, the stimuli of negative doctor–patient relationships elicited a larger amplitude in the 500–800 ms time window (Fig. 3) compared to the neutral ones, thereby indicating that the participants had completed the perception. This phenomenon might be attributed to our experimental materials, that is, the stimuli of negative relationships, which showed physical conflicts between doctors and patients and even displayed threatening situations with knives and blood [49], while no physical threat was shown in neutral stimuli [7, 38, 55]. Wheaton et al. [53] found that individuals paid attention to stimuli that had clear threats, and stimuli of negative doctor–patient relationships, including knives, blood, and physical conflict, would make them feel threatened. Hence, when patients are confronted with the stimuli of negative doctor–patient relationships, their LPP amplitude is affected by the threat elements in them.

In addition, no significant difference in LPP was observed in the 800–1100 ms time window (Fig. 3). The LPP in neutral and emotional stimuli was indistinct when individuals processed implicit emotional regulation [18, 45]. The main reason for this finding was that the participants spontaneously engaged in implicit emotion regulation [45], specifically, the instructions did not implicitly tell the participants to use emotional strategies to cope with the stimuli. However, some scholars believe that participants used implicit emotional regulation spontaneously to protect themselves when confronted with a series of medical events with threat elements, which was conducive to their survival [7, 34].

The LPP in the stimuli of neutral doctor–patient relationships in the 1100–1500 ms time window was significantly higher than that in the positive stimuli, and no significant difference was observed with the negative stimuli (Fig. 3). This result was different from our hypothesis, as previous studies have shown that individuals might have a larger LPP amplitude when they see stimuli that generate strong aversion [18, 21]. In addition, the importance of emotions concerning LPP can be evaluated precisely when individuals perceive these stimuli, which require high involvement and involve the extraction of past experiences [23, 32]. Therefore, the stimuli of neutral doctor–patient relationships elicited a larger LPP in the participants who played the role of patients according to the instructions, perhaps because they extracted their past experiences of seeing a doctor. This assumption was confirmed by the imaginings of the participants before the experiment as well as their self-reports after the experiment. In their imagination, most participants expected doctors to be friendly, especially when they paid a considerable amount of money to see them (or waited in line for a long time). However, in Chinese hospitals, the clinical reception mode of doctors assumes the form of “a doctor with numerous patients” [51, 54], which requires doctors to work with dozens of patients in a short period; thus, they might not engage in many positive nonverbal behaviors with all their patients (e.g., gentle eye contact). However, if the doctors’ performance is negative, patients would doubt the doctor–patient relationship [41]. The self-report showed that patients felt dissatisfied when they saw a doctor with an expressionless face when interacting with them.

Patient awareness was reflected in the 1100–1500 ms time window of the LPP component. That is, although the stimuli of negative doctor–patient relationships symbolized threatening information (e.g., knives, blood), such events were infrequent for most patients [30, 38, 48], by contrast, expressionless and apathetic doctors in neutral doctor–patient relationships were ubiquitous for patients, which made them feel unsatisfied and evoked lasting attention. The motivated attention theory posits that these previous negative personal experiences act as salient motivation to elicit lasting attention for the stimulation of neutral experiences in patients. These findings are consistent with previous studies on motivated attention, by which individuals pay more attention to stimuli that could generate their motivation. Even though most patients perceived the stimuli of negative doctor–patient relationships as the most negative, personal experience led them to pay attention to the neutral ones, and they had reactions that were intense, persistent, and difficult to eliminate by spontaneous emotion regulation strategies. On the contrary, the negative stimuli elicited a response to a certain extent, but most participants did not have the same personal experience; therefore, sensitivity decreased for a while.

Our study has several limitations. First, we only explored the perspective of patients in the doctor–patient relationship and ignored that of doctors. Therefore, we could explore doctors’ views of doctor–patient relationships as well as the influence of their perceptions. Further, although we tried our best to encourage the participants who had had clinical experience in the previous 6 months to play the role of patients in the experiment, we could not control the distinction between them and real patients who were under treatment as the latter might have profound experiences of seeing a doctor or experiencing a disease. Thus, future research could include multiple perspectives, conduct research in a real environment, and divide the negative stimuli of doctor–patient relationships into two types, patient-induced negative stimuli and doctor–induced negative stimuli, to explore the relationship between doctors and patients more fully.

Despite these limitations, the current study found that patients’ perceptions of the stimuli of doctor–patient relationships had different valence from the EEG level. We found that people prioritize positive stimuli in doctor–patient relationships compared with neutral ones. Although negative stimuli presented explicit threat cues to patients, they did not elicit lasting attention. In the long term, when patients perceived neutral doctor–patient relationships because of their past experiences, the neutral stimuli would capture more lasting attention. These results not only provide reliable evidence that doctors’ nonverbal behaviors affect patients’ cognition of doctor–patient relationships, but they also indicate that most task-oriented communication methods of doctors at work could not meet patients’ expectations. In future research, we could explore the doctor–patient relationship from this perspective and provide a basis for improving the current Chinese medical model and the strained doctor–patient relationship.

## Conclusion

In summary, patients paid more attention to the stimuli of positive doctor–patient relationships because they were expected to have a positive doctor–patient relationship. Although the threatening elements in negative stimuli would catch patients’ attention and make them have implicit emotional regulation, neutral stimuli involving poker-faced doctors would cause lasting attention. These results illustrate the patients’ real perception of the different valence of doctor–patient relationship stimuli.

## Supplementary Information

**Additional file 1: Fig S1**. The brain topography maps in P2. The difference waves in positive-neutral in Pz, Fz, and Cz.

## Data Availability

The datasets used and/or analyzed in this study are available from the corresponding author on reasonable request.
