# Supplementary figures and images for: Chinese patients’ response to doctor–patient relationship stimuli: evidence from an event-related potential study
Source: BMC Psychol. 2022 Nov 5;10:253. doi: 10.1186/s40359-022-00961-y (PMC9636646; doi:10.1186/s40359-022-00961-y)

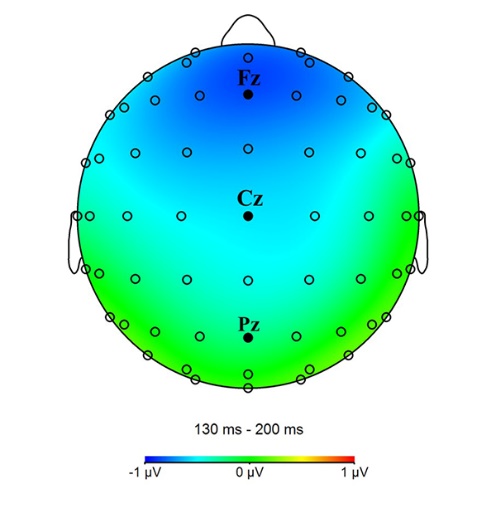


Fig S1. The brain topography maps in P2. The difference waves in Positive-Neutral in Pz, Fz, and Cz.

Supplement: Supplementary file 1 — Additional file 1: Fig S1. The brain topography maps in P2. The difference waves in positive-neutral in Pz, Fz, and Cz. [file 40359_2022_961_MOESM1_ESM.docx]
